# The serine-rich repeat glycoprotein Srr2 mediates *Streptococcus agalactiae* interaction with host fibronectin

**DOI:** 10.1186/s12866-024-03374-6

**Published:** 2024-06-22

**Authors:** Angelica Pellegrini, Chiara Motta, Elisa Bellan Menegussi, Andrea Pierangelini, Simona Viglio, Francesco Coppolino, Concetta Beninati, Vincenzo De Filippis, Giulia Barbieri, Giampiero Pietrocola

**Affiliations:** 1 Department of Molecular Medicine, University of Pavia, Pavia, Italy; 2 Department of Pharmaceutical and Pharmacological Sciences, University of Padua, Padua, Italy; 3 Department of Human Pathology and Medicine, University of Messina, Messina, Italy; 4 Department of Biology and Biotechnology “Lazzaro Spallanzani”, University of Pavia, Pavia, Italy

**Keywords:** Fibronectin, Bacterial adhesins, Host-pathogen interaction, Srr2 adhesin, Streptococcus agalactiae, DLL binding mechanism

## Abstract

**Background:**

Group B Streptococcus (GBS) is a commensal of healthy adults and an important pathogen in newborns, the elderly and immunocompromised individuals. GBS displays several virulence factors that promote colonisation and host infection, including the ST-17 strain-specific adhesin Srr2, previously characterised for its binding to fibrinogen. Another common target for bacterial adhesins and for host colonization is fibronectin, a multi-domain glycoprotein found ubiquitously in body fluids, in the extracellular matrix and on the surface of cells.

**Results:**

In this study, fibronectin was identified as a novel ligand for the Srr2 adhesin of GBS. A derivative of the ST-17 strain BM110 overexpressing the *srr2* gene showed an increased ability to bind fibrinogen and fibronectin, compared to the isogenic wild-type strain. Conversely, the deletion of srr2 impaired bacterial adhesion to both ligands. ELISA assays and surface plasmon resonance studies using the recombinant binding region (BR) form of Srr2 confirmed a direct interaction with fibronectin with an estimated Kd of 92 nM. Srr2-BR variants defective in fibrinogen binding also exhibited no interaction with fibronectin, suggesting that Srr2 binds this ligand through the dock-lock-latch mechanism, previously described for fibrinogen binding. The fibronectin site responsible for recombinant Srr2-BR binding was identified and localised in the central cell-binding domain of the protein. Finally, in the presence of fibronectin, the ability of a Δ*srr2* mutant to adhere to human cervico-vaginal epithelial cells was significantly lower than that of the wild-type strain.

**Conclusion:**

By combining genetic and biochemical approaches, we demonstrate a new role for Srr2, namely interacting with fibronectin. We characterised the molecular mechanism of this interaction and demonstrated that it plays a role in promoting the adhesion of GBS to human cervico-vaginal epithelial cells, further substantiating the role of Srr2 as a factor responsible for the hypervirulence of GBS ST-17 strains. The discovery of the previously undescribed interaction between Srr2 and fibronectin establishes this adhesin as a key factor for GBS colonisation of host tissues.

**Supplementary Information:**

The online version contains supplementary material available at 10.1186/s12866-024-03374-6.

## Introduction

*Streptococcus agalactiae*, commonly known as Group B *Streptococcus* (GBS), is frequently found as an asymptomatic colonizer of the human gastrointestinal and genital tract. However, under certain conditions, it can act as an opportunistic pathogen, leading to severe infections in newborns, the elderly, and immunocompromised individuals [1]. The most common clinical manifestations of GBS infections in infants include meningitis and sepsis, whereas in adults, they manifest as bacteremia, soft tissue infections, and pneumonia [2].

The ability of GBS to breach host barriers is given by virulence factors exposed on its bacterial surface and conferring the ability to evade the immune system, colonize, invade and damage host’s tissues [3]. In this context, adhesins play a key role in mediating the interaction of GBS with host cells, plasma proteins (such as fibrinogen, Fbg, and plasminogen, Plg), and components of the extracellular matrix (ECM), such as laminin and fibronectin (Fn) [3].

Notably, the expression of two GBS surface proteins, HvgA and Srr2, encoded exclusively in strains belonging to the sequence-type 17 (ST-17) [4, 5], was demonstrated to contribute to the hyper-virulence of these isolates, which strongly associate with the development of Late-Onset Disease (LOD) and meningitis in newborns [6, 7]. Non-ST-17 strains express other allelic variants of these proteins [8].

**Fig. 1:**
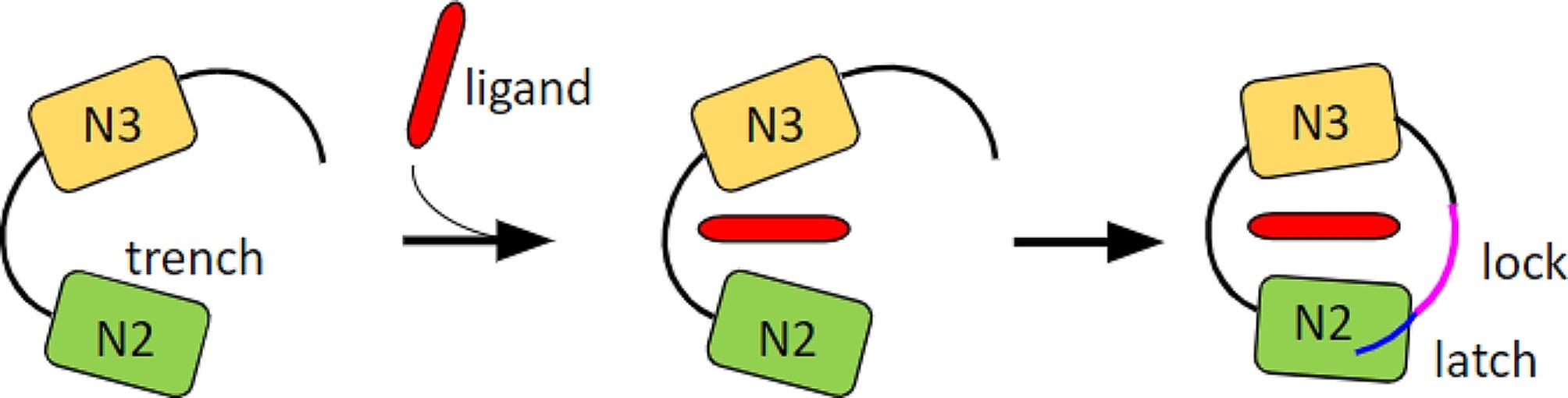
Schematic representation of the dock, lock, and latch (DLL) mechanism of binding. According to the DLL mechanism, the ligand binding takes place in multiple steps: the ligand first docks into the trench (N2 subdomain), followed by conformational change and redirection of the carboxy-terminal extension of the N3 subdomain (latch), so that amino acid residues in this extension crossover the binding trench and lock the bound ligand in place

Serine-rich repeat (Srr) proteins, such as Srr1 in non-ST-17 strains and Srr2 in hypervirulent ST-17 strains, have a similar structure, comprising a signal sequence, a serine-rich region (SRR), a binding region (BR) for ligand attachment, a second SRR region, and an LPXTG motif anchoring the C terminus to the cell wall [9]. Both proteins display the ability to bind the plasma glycoprotein Fbg through their BR domain [9, 10]. The binding mechanism employed is defined as “dock-lock-latch” (DLL), whereby Fbg binds between the N2 and N3 domains of the BR, inducing a conformational change in the C-terminal portion of the N3 domain (latch), which folds to form a β-sheet and interacts with the N2 domain (Fig. 1). The ligand is thus locked at the BR [9, 11].

Besides binding to Fbg, Srr1 facilitates GBS interaction with cytokeratin 4, contributing to colonization of the female genital tract [12].

In contrast, Srr2 enhances GBS virulence by binding to Fbg, plasmin, and Plg, disrupting the host coagulation system and promoting bacterial spread [11].

Srr2 also interacts with eukaryotic receptors α5β1 and αvβ3, overexpressed in cerebral vessels, enhancing adhesion of ST-17 strains to brain endothelial cells and facilitating blood brain barrier (BBB) invasion, especially in newborns [13]. Crossing the BBB can occur transcellularly or paracellularly, modifying endothelial cell permeability via integrins binding [13].

Srr2 also enables GBS ST-17 translocation across the intestinal epithelial barrier via transcytosis of Peyer’s patch M cells, leading to neonatal LODs [7]. Hormones estradiol (E2) and progesterone (P4), which accumulate in the fetus during pregnancy and are abundant in the first months of newborns’ lives, increase barrier permeability, promote M cell maturation and enhance Srr2-mediated GBS crossing of the intestinal barrier, thereby favouring concentrations, its access to the lymphatic system and dissemination throughout the body [7].

The expression of *srr2* is controlled by two regulatory systems: the two component-system CovRS and the global transcriptional regulator CodY [14, 15]. The response regulator CovR is a global repressor of virulence-associated genes whose activity is dependent on its cognate histidine kinase CovS and on at least two associated regulators, Abx1 and Stk1 [16, 17]. CodY is a branched-chain amino acids dependent regulator which acts mainly as a repressor of GBS gene expression, controlling almost 13% of the genome of the bacterium. Its activity is required for GBS virulence in vivo, as a *codY*-null strain displays a reduced ability to cause infections in neonatal and adult mice [15]. Deletion of *codY* or *covR* in the hypervirulent GBS strain BM110 (ST-17) resulted in *srr2* overexpression, demonstrating that both regulators act as repressors of this gene [14, 15].

With the objective of elucidating the mechanisms by which CodY influences GBS virulence and considering the important contribution of interactions with plasma and ECM proteins to the infection process, in the present study we compared the adhesive properties of a *codY*-null and wild-type ST-17 strain (BM110) to fibronectin and fibrinogen and investigated the role played by Srr2 in such interactions. Furthermore, we report a new ligand for this multivalent adhesin and describe for the first time the interaction of an adhesin with fibronectin via the DLL mechanism. We also investigate the functional implications of this interaction concerning the adhesive properties of GBS to host cells.

## Materials and methods

### Bacterial strains and culture conditions

All strains used in this study are listed in Table 1. GBS cells were grown overnight in Todd-Hewitt (TH) medium supplemented with yeast extract (THY) (SIGMA) at 37 °C. For ELISA assays involving bacterial adhesion to immobilized proteins, bacteria were harvested from the cultures by centrifugation, washed, and suspended in phosphate-buffered saline (PBS, NaCl 130 mM, NaH_2_HPO_4_ 7 mM, NaH_2_PO_4_ 3 mM, pH 7.4) at an optical density at 600 nm (OD_600_) of 1.0. *Escherichia coli* XL1-Blue (Agilent Technologies, CA, USA) or BL21 (DE3) (Agilent Technologies, CA, USA) strains transformed with vector pET28a (Stratagene, La Jolla, CA) or derivatives were grown in Luria agar and Luria broth (VWR) containing 50 μg/ml kanamycin at 37 °C with shaking. Erythromycin (Sigma-Aldrich) was used at 10 μg/ml for selection and maintenance of plasmid pG1 and its derivatives in GBS.

### Plasmids and strains construction

Oligonucleotides and plasmids used in this work are listed in Tables 2 and 3, respectively.

To prepare plasmid pET28a-*srr2*-BR, a 1056 bp fragment encoding amino acids 192–543 of Srr2 was PCR amplified with primers srr2192F and srr2BRRev using the BM110 chromosomal DNA as template. The obtained amplicon was inserted by Gibson assembly (NEBuilder HiFi DNA Assembly Cloning Kit, New England Biolabs) between the *Bam*HI and *Eco*RI restriction sites of plasmid pET28a. The ∆latch variant (amino acids 192–530) was obtained by the same approach, using srr2BR_latchR as reverse primer. Plasmid pET28a-*srr2*-BR-∆trench, containing substitution and deletion mutations in the trench sequence [11], was constructed by producing two PCR fragments: an amplicon containing the 5’ part of the sequence encoding the Srr2-BR was synthesized by using oligonucleotide srr2192F as the forward primer and mutagenic oligonucleotide trenchR as the reverse primer; a fragment containing the 3’ part of the sequence encoding the Srr2BR was synthesized by using the mutagenic forward oligonucleotide trenchF and the reverse primer srr2BRRev. The PCR products were fused and cloned into pET28a by Gibson assembly. The DNA fragments encoding the N2 and N3 subdomains of Srr2-BR were PCR amplified from the genome of the GBS strain BM110 using the Srr2-BR_N2For - Srr2-BR_N2Rev primers for N2 region and Srr2-BR_N3For - Srr2-BR_N3Rev primers for N3 region. Amplified N2 and N3 fragments were cloned into the expression plasmid pET28a between the BamHI and SalI restriction sites, generating constructs pET28a-*srr2*-BR_N2 and pET28a-*srr2*-BR_N3, respectively. All constructs were verified by sequencing.

The BM110 ∆*srr2* mutant was constructed by allelic replacement using the thermosensitive pG1 vector (kind gift from Arnaud Firon, Institut Pasteur, France). An in-frame deletion of 3429 nucleotides was created within the *srr2* gene, corresponding to the sequence encoding amino acids 34 -1176. The upstream (717 bp) and downstream (567 bp) genomic regions flanking the target sequence were PCR amplified using the primers pairs pG1srr2UpF/pG1srr2UpR and pG1srr2DwF/pG1srr2DwR, respectively. The two PCR fragments were fused and cloned by Gibson assembly into plasmid pG1, previously linearized by PCR using the divergent primers pG1F and pG1R. The obtained pG1-∆*srr2* plasmid was used to electroporate BM110 and BM110 ∆*codY* electrocompetent cells and transformants were selected at permissive temperature (30 °C) with erythromycin. After chromosomal integration at the target locus at restrictive temperature (37 °C), de-recombination and loss of the deletion vector were obtained by growth at 30 °C without selective pressure. The final step can result in a back to WT (BTWT) allele (*srr2* BTWT) or in a strain carrying the desired deletion (BM110 ∆*srr2;* BM110 ∆*codY* ∆*srr2*). The BTWT or deletion genotypes were discriminated by PCR (with external primers secY2F and srr2Up1) and Sanger sequencing.

### Expression and purification of recombinant proteins

Recombinant proteins Srr2-BR, Srr2-BR ∆latch, Srr2-BR ∆trench, Srr2-BR-N2 or N3 subdomain were expressed with His_6_ C-terminal tags using pET28a vector (Qiagen, Hilden, Germany) in *E. coli* BL21 DE3 cells (Agilent Technologies, CA, USA). Overnight starter cultures were diluted 1:100 in Luria broth containing kanamycin (50 μg/ml) and incubated with shaking until the culture reached the exponential phase (OD_600_ of 0.6). Recombinant protein expression was induced by the addition of 1 mM (final concentration) isopropyl 1-thio-β-D-galactopyranoside (IPTG) (Thermo, Italy) to the culture. After overnight shaking at 20 °C, bacterial cells were harvested by centrifugation and resuspended in lysis buffer (50 mM NaH_2_PO_4_, 300 mM NaCl, pH 7.4, containing 1 mM Phenylmethanesulfonyl fluoride (PMSF) (Sigma-Aldrich) as the protease inhibitor, 20 μg/ml DNase (Sigma-Aldrich), MgCl_2_ (1 mM)), and lysed by sonication (70% amplitude, 12 × 30″ on/off, 1′30″ interval between sonication steps). The cell debris was removed by centrifugation, and proteins were purified from the supernatants by Ni^2+^-affinity chromatography on a HiTrap chelating column (GE Healthcare, Buckinghamshire, UK). Protein purity was assessed by 12.5% SDS-PAGE and Bio-Safe Coomassie staining (BioRad, Hercules, CA, USA). A bicinchoninic acid protein assay (Pierce, Rockford, IL, USA) was used to measure the concentration of purified proteins.

### Reagents, proteins, and antibodies

Bovine serum albumin (BSA), human Fbg, human collagen, protease-free DNase I, skim milk, trypsin, and HRP-conjugated secondary anti-mouse and anti-rabbit IgG were purchased from Sigma-Aldrich. The anti-Fn antibody, anti-Fbg antibody and anti-Srr2 antibody were raised in mice by a routine immunization procedure using purified Fn or Fbg or Srr2-BR as antigens, respectively. Briefly, the polyclonal antibodies were produced by immunizing 6-week-old specific pathogen-free CD1 mice (Charles River Laboratories, Italia) by the intraperitoneal injection of 50 μg of the antigen in alum on days 0, 14, and 28. Sera were collected 15 days after the last immunization. The anti-GBS antibody was raised in rabbit using 50 μg of heat-inactivated GBS bacterial cells as antigen as reported above. Animals were sacrificed by cervical dislocation after sedation with carbon dioxide. The polyclonal antibodies production method was carried out in accordance with relevant guidelines and regulations; all experimental protocols were approved by the Ethics Committee of the University of Pavia (OPBA permit n° 1,909,213) and of the University of Messina (OPBA permit n° 1,805,210) and by the Ministero della Salute of Italy (permit no. 665/2015). The study is reported in accordance with ARRIVE guidelines (https://arriveguidelines.org). Rabbit anti-mouse and goat anti-rabbit horseradish peroxidase (HRP)-conjugated secondary antibodies were purchased from Dako Cytomation (Glostrup, Denmark). OPD tablets (o-phenylenediamine dihydrochloride) were purchased by ThermoScientific (Rockford, IL, USA). Fn was purified from plasma through gelatin-Sepharose affinity chromatography as previously described [18]. Freshly drawn blood to prepare human plasma was obtained from healthy volunteers with informed consent and permission of the ethical board of the University of Pavia (permit no. 19,092,019). The research abides by the Declaration of Helsinki principles. Plasma fractions were frozen in aliquots after blood centrifugation and stored at − 20 °C. Fn fragments were previously obtained by fractionation of thermolysin-digested plasma Fn on ion-exchange/gel filtration chromatography column [18].

### ELISA-type solid-phase binding assays

#### Fn or Fbg binding by GBS strains

The ability of bacteria to interact with immobilized human Fn or Fbg was evaluated by ELISA-based assay. Microtiter wells were coated overnight at 4 °C at the indicated concentrations of Fn or FBG in 0.1 M sodium carbonate, pH 9.5. The plates were washed three times with 0.5% (v/v) Tween 20 in PBS (PBST). To block additional protein-binding sites, wells were treated for 1 h at room temperature (RT) with 200 μl of 2% BSA in PBS. Bacterial adhesion was evaluated by incubation of 100 μl/well bacterial cells (OD_600nm_ = 1.0) in PBS for 1 h at 37 °C. Attached bacteria were detected through rabbit anti-GBS antibody (1 μg/well) followed by peroxidase-conjugated secondary goat anti-rabbit IgG diluted 1:1000. After washing, OPD was added and the absorbance at 490 nm was measured by using an ELISA plate reader (BMG Labtech).

#### Binding of rSrr2-BR, rSrr2-BR ∆latch, rSrr2-BR ∆trench, rSrr2-N2 or rSrr2-N3 subdomains to Fn and FBG

The ability of soluble human Fn or Fbg to bind to immobilized recombinant proteins (rSrr2-BR, rSrr2-BR ∆latch, rSrr2-BR ∆trench, rSrr2-N2 or rSrr2-N3 subdomains) was determined using ELISA assay. Microtiter wells were coated overnight at 4°C with 1 μg/well of each recombinant protein in 0.1 M sodium carbonate, pH 9.5. After 1 h treatment at RT with BSA, the plates were incubated for 1 h with increasing amounts of Fn (up to 240 nM) or Fbg (up to 7.5 nM). Mouse anti-Fn IgG (1:4000) or anti-Fbg (1:1000) in 1% BSA was added to the wells, followed by incubation for 60 min. After washing, the plates were incubated for 45’ with peroxidase-conjugated secondary rabbit anti-mouse IgG diluted 1:1000. After washing, OPD was added and the absorbance at 490 nm was determined.

The ability of soluble rSrr2-BR to bind to immobilized Fn was also determined. Microtiter wells were coated overnight at 4°C with 1 μg/well of Fn in 0.1 M sodium carbonate, pH 9.5. After 1 h treatment at RT with BSA, the plates were incubated for 1 h with increasing amounts of rSrr2-BR (up to 2.5 μM). Mouse anti-Srr2 IgG (1:4000) in 1% BSA was added to the wells, followed by incubation for 60 min. After washing, the plates were incubated for 45’ with peroxidase-conjugated secondary rabbit anti-mouse IgG diluted 1:1000. After washing, OPD was added and the absorbance at 490 nm was determined.

#### Inhibition - competition assays

To assess the effect of sodium chloride on rSrr2-BR / Fn or rSrr2-BR / Fbg interaction, microtiter wells coated with 1 μg of rSrr2-BR were incubated with (5 μg) of Fn or (0.25 μg) of Fbg in presence of increasing concentrations of salt (from 0 to 1 M). After washing, complex formation was detected by incubation of the wells with a mouse anti-Fn (1:4000) or anti-Fbg (1:1000) IgG followed by peroxidase-conjugated secondary rabbit anti-mouse IgG diluted 1:1000. After washing, OPD was added and the absorbance at 490 nm was determined.

The binding of 1 μg of Fn to surface coated rSrr2-BR (1 μg/well) in presence of equimolar increasing concentrations (up to 300 nM) of Fbg or collagen was detected with anti-Fn antibody as reported above.

To determine the effect of soluble CBD fragment on the rSrr2-BR / Fn interaction, microtiter wells, coated with 1 μg of Fn, were incubated with 1 μg of rSrr2-BR in the presence of increasing concentration of CBD fragment (up to 10 μg/well). rSrr2-BR binding to Fn was detected with mouse anti-Srr2 antibody (1:4000) followed by peroxidase-conjugated secondary rabbit anti-mouse IgG diluted 1:1000. After washing, OPD was added and the absorbance at 490 nm was determined.

#### Detection of Srr2-expression on BM110 cells during bacterial growth phases

The expression of Srr2 on the surface of BM110 cells was assessed through ELISA-based assay. Evaluation was performed by harvesting bacteria by centrifugation at different time points of growth and immobilizing 100 μl of cells resuspended in PBS at OD_600_ = 1 per well (10^7^ cells/well), overnight at 37 °C. Protein expression was detected using the mouse anti-Srr2 antibody (1:4000), followed by HRP conjugated rabbit anti-mouse, OPD and absorbance reading at 490 nm. To confirm that the same amount of BM110 cells were immobilised at the different time points, control wells were seeded and incubated with the anti-GBS antibody. The complex was detected as reported above (Fig. S1). To exclude the possibility that the reactivity could be due to the expression of Ig-binding proteins on the bacterial surface, the assay was conducted in parallel by incubating the wells with an isotype control antibody (data not shown).

### Surface plasmon resonance

The binding of Fn to Srr2 proteins was evaluated by Surface Plasmon Resonance (SPR). SPR analyses were performed on a Biacore X-100 dual flow-cell instrument (GE Healthcare, Chicago, IL, USA). Purified rSrr2 proteins (50 μg/mL) were covalently immobilized at pH 4.75 on a carboxymethylated-dextran chip (CM5) using the amine coupling chemistry. All measurements were carried out at 25 °C in HEPES-EP + buffer (10 mM HEPES, pH 7.4, 0.15 M NaCl, 50 mM EDTA, 0.005% v/v polyoxyethylene sorbitan) at a flow rate of 30 μL/min. Each sensogram was subtracted for the corresponding baseline, obtained on the reference flow cell and accounting for nonspecific binding. The dissociation constants (*K*_d_) of the Fn-Srr2 interaction were obtained as a fitting parameter by plotting the RU values at the steady state (RU_eq_) *versus* [Fn] and interpolating the data points with Eq. 1, describing a 1:1 binding model [19, 20]

1 $$R{U_{eq}} = R{U_{max}} \cdot \frac{{\left[ L \right]}}{{{K_d} + \left[ L \right]}}$$

where L is the concentration of Fn, while RU_eq_ and RU_max_ are the RU values measured at the steady state with intermediate or saturating [L], respectively.

### Dot-blot and western-blot analyses

Binding of rSrr2-BR to Fn or its fragments was evaluated through Dot-blot and Western-blot analyses. For dot-blot assay, equimolar concentrations of Fn or its fragments were dotted onto a PVDF membrane (BioRad). After overnight incubation at 4 °C with 5% skim milk (w/v) in 0.5% (v/v) Tween 20 in PBS (PBST), the membrane was treated for 1 h at 22 °C with 1 μg/mL of rSrr2-BR. The membrane was incubated with a mouse polyclonal anti-Srr2 antibody (1:2000) in 2% (w/v) skim milk for 1 h at 22 °C. Following several washings with PBST, the membrane was treated with 45 min at 22 °C with an HRP-conjugated rabbit anti-mouse IgG (1:10000) in 2% (w/v) skim milk. Dot-blot was developed using the Westar Supernova detection kit (Cyanagen srl, Bologna, Italy), and ImageQuant™ LAS 4000 mini-biomolecular imager (GE Healthcare) was used to capture images of the spots. For Western-blot analysis, rSrr2-BR was covalently coupled to Carboxyl (COOH) microparticles through the PolyLink Protein Coupling Kit for COOH Microspheres (Polysciences, Germany). Srr2-coupled particles were incubated for 1 h with equimolar concentrations of Fn or its fragments to allow binding. After washings with PBS, proteins bound to microparticles were dissociated through incubation with SDS-containing loading buffer and the samples were subjected to SDS-PAGE and transferred to a PDVF membrane. After overnight incubation at 4 °C with 5% skim milk in PBST, the membrane was treated for 1 h at 22 °C with mouse polyclonal anti-Fn antibody (1:4000) in 2% (w/v) skim milk for 1 h at 22 °C. After washing, the membrane was treated with 45 min at 22 °C with an HRP-conjugated rabbit anti-mouse IgG (1:10000) in 2% (w/v) skim milk and bands developed using the Westar Supernova detection kit (Cyanagen srl, Bologna, Italy). Signal was detected through an ImageQuant™ LAS 4000 mini-biomolecular imager (GE Healthcare).

### Epithelial cell culture and cell adhesion assays

Epithelial cervico-vaginal HeLa cells were seeded at 1.5 × 10^5^ cell density per well in 24-well tissue culture plates in Dulbecco’s modified Eagle’s medium (DMEM) supplemented with 10% Fn- depleted fetal bovine serum (FBS) and allowed to attach for 24 h at 37°C in 5% CO_2_. The effect of Fn on bacterial adhesion/invasion to monolayers was tested by pre-incubating the cells with 20 μg/ml of Fn for 1 h prior to infection. GBS cells grown to the late exponential phase and resuspended in PBS were used to infect the cell monolayers with a multiplicity of infection (MOI) of 10. After a 30’ incubation, monolayers were washed three times with PBS to remove the non-adherent bacteria, lysed using 500 μl of cold 1% Triton X-100, and serial dilutions of the cell lysates were plated, and CFU were counted after incubation. Percent of adhesion of each strain was calculated as follows (number of CFUs on plate)/(number of CFUs of initial inoculum) × 100. To enumerate internalized bacteria, 30’ after infection the monolayers were furtherly incubated for 1 h in medium supplemented with 100 μg/ml penicillin/streptomycin to kill adhered extracellular bacteria. Following incubation, monolayers were washed three times with PBS, lysed and plated to enumerate cell-internalised bacteria.

### Statistical methods

Analyses were performed using Prism 9.0 (GraphPad). A minimum of three biological replicates (three independent experiments) were conducted for each experiment. Comparison of two groups was conducted through the two-tailed Student’s t-test. The one-way ANOVA test was used to compare more than two groups, in combination with a post- hoc test (Dunnett’s multiple comparison test or Tukey’s honest significant test). *P* values < 0.05 were considered statistically significant.

**Table 1:** List of bacterial strains used in this study

Bacterial strain	Relevant properties	Reference
*S. agalactiae*		
BM110	Serotype III, ST-17, human hypervirulent clinical isolate	[4]
BM110 ∆*codY*	BM110 carrying an in-frame *codY* deletion	[15]
BM110 ∆*srr2*	BM110 carrying an in-frame *srr2* deletion	This study
BM110 ∆*srr2* (*srr2* BTWT)	BM110 ∆*srr2* with genome complemented to wild-type	This study
BM110 ∆*codY* ∆*srr2*	BM110 carrying in-frame deletions of *srr2* and *codY* genes	This study
BM110 ∆*codY* ∆*srr2 (srr2* BTWT)	BM110 ∆*codY* ∆*srr2* with genome complemented to wild-type for *srr2* gene	This study
NEM316	Serotype III, ST-23, human clinical isolate	[21]
A909	Serotype Ia, ST-7, human isolate	[8]
COH1	Serotype III, ST-17, human hypervirulent clinical isolate	[8]
*E. coli*		
XL-1blue	*E. coli* cloning host	Agilent Technologies, CA, USA
BL21 (DE3)	*E. coli* cloning host	Agilent Technologies, CA, USA

**Table 2:** List of primers used in this study

Primer	5’ – 3’ sequence
srr2192F	GTGGACAGCAAATGGGTCGCGGATCCGAAGCGGCAACGACCGCTAGAGTTC
srr2BRRev	AAGCTTGTCGACGGAGCTCGAATTCTCATTGAGCATTTACATCTGAATATCCCGATTCAG
srr2BR_latchR	AAGCTTGTCGACGGAGCTCGAATTCTCAAGTATGATTATGTTCTACACCATTGTTC
trenchR	GGCACGCCCGGGTGCTGCCGCTTGAGAATCATAAATTCCAGTCGC
trenchF	GCGGCAGCACCCGGGCGTGCCGCGGCTTCAAAAAATAATATT
pG1srr2UpF	CTGAAGCCGAGTTATCACCTGTCATAACACTTTTGACCCAGTG
pG1srr2UpR	GGAAACAGCTATGACCATGATTACGAATTCGAGAGCGGCTATTTATTTTTAG
pG1srr2DwF	GCATGCCTGCAGGTCGACTCTAGAGGATCCGAAACACCATATGCTTGTATAAC
pG1srr2DwR	CACTGGGTCAAAAGTGTTATGACAGGTGATAACTCGGCTTCAG
pG1F	GAGCTCGGTACCCGGGGA
pG1R	GAATTCGTAATCATGGTCATAG
secY2F	CAGTGGATAATAAAACACGTTGTCC
srr2Up1	GATAGGAGGGGTCTTTATAGGC
Srr2-BR_N2For	TCACGGATCCGAAGCGGCAACGACC
Srr2-BR_N2Rev	ACGTCGACTCATTTCACTGTTTCTTTAATTGTAGC
Srr2-BR_N3For	TCACGGATCCGATCCACCGGTAAGAATTGATTTTG
Srr2-BR_N3Rev	ACGTCGACTCATTGAGCATTTACATCTGAATATCC

**Table 3:** List of plasmids used in this study

Plasmid	Resistance	Relevant properties	Reference
pG1	Erythromycin	Plasmid containing a temperature sensitive origin of replication for GBS and a ColE1 origin of replication for *E. coli*.	[22]
pG1-∆*srr2*	Erythromycin	pG1 carrying the *srr2* deletion cassette	This study
pET28a	Kanamycin		Qiagen
pET28a-srr2-BR	Kanamycin	pET28a carrying the wild-type Srr2 binding region ORF	This study
pET28a-srr2-BR ∆latch	Kanamycin	pET28a carrying a mutated Srr2 binding region	This study
pET28a-srr2-BR∆trench	Kanamycin	pET28a carrying a mutated Srr2 binding region	This study
pET28a-srr2-BR_N2	Kanamycin	pET28a carrying the N2 subdomain of Srr2-BR	This study
pET28a-srr2-BR_N3	Kanamycin	pET28a carrying the N3 subdomain of Srr2-BR	This study

## Results

### Srr2 promotes specific binding of GBS ST-17 strains to Fn

We have recently shown that deletion of the gene encoding the global transcriptional regulator CodY in the hypervirulent GBS strain BM110 (∆*codY*) results in a twelve-fold increase in *srr2* transcript abundance during exponential growth in liquid TH medium, compared to the wild-type strain [15]. As the ability of CodY to bind DNA is increased by its interaction with branched-chain amino acids, the regulatory capacity of this protein is dependent on the abundance of these cofactors.

**Fig. 2:**
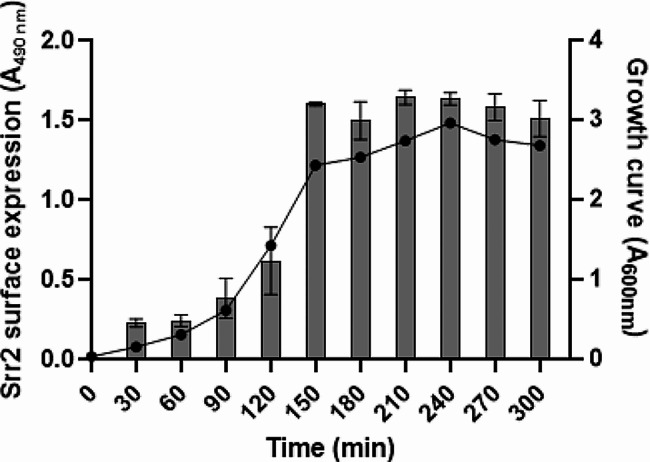
Srr2 expression on GBS cell-surface increases during bacterial growth. At the indicated times, bacterial cells were collected, washed and then immobilized onto microtiter wells. The presence of Srr2 on cell surface was revealed using anti-Srr2 IgG followed by secondary HRP-conjugated IgG (left axis). Bacterial growth curve is also reported (right axis). The data points are the means ± SD from three independent experiments, each performed in triplicate

To assess whether the levels of this adhesin vary in response to the bacterial growth conditions, Srr2 protein levels were evaluated by an ELISA assay performed using an anti-Srr2 antibody on immobilized BM110 wild-type cells collected at 30-min intervals during growth in TH medium. A sharp increase in protein levels was observed at the beginning of the stationary phase, starting at T0 (transition point from the exponential to stationary growth phase, estimated at 150 min) (Fig. 2).

**Fig. 3:**
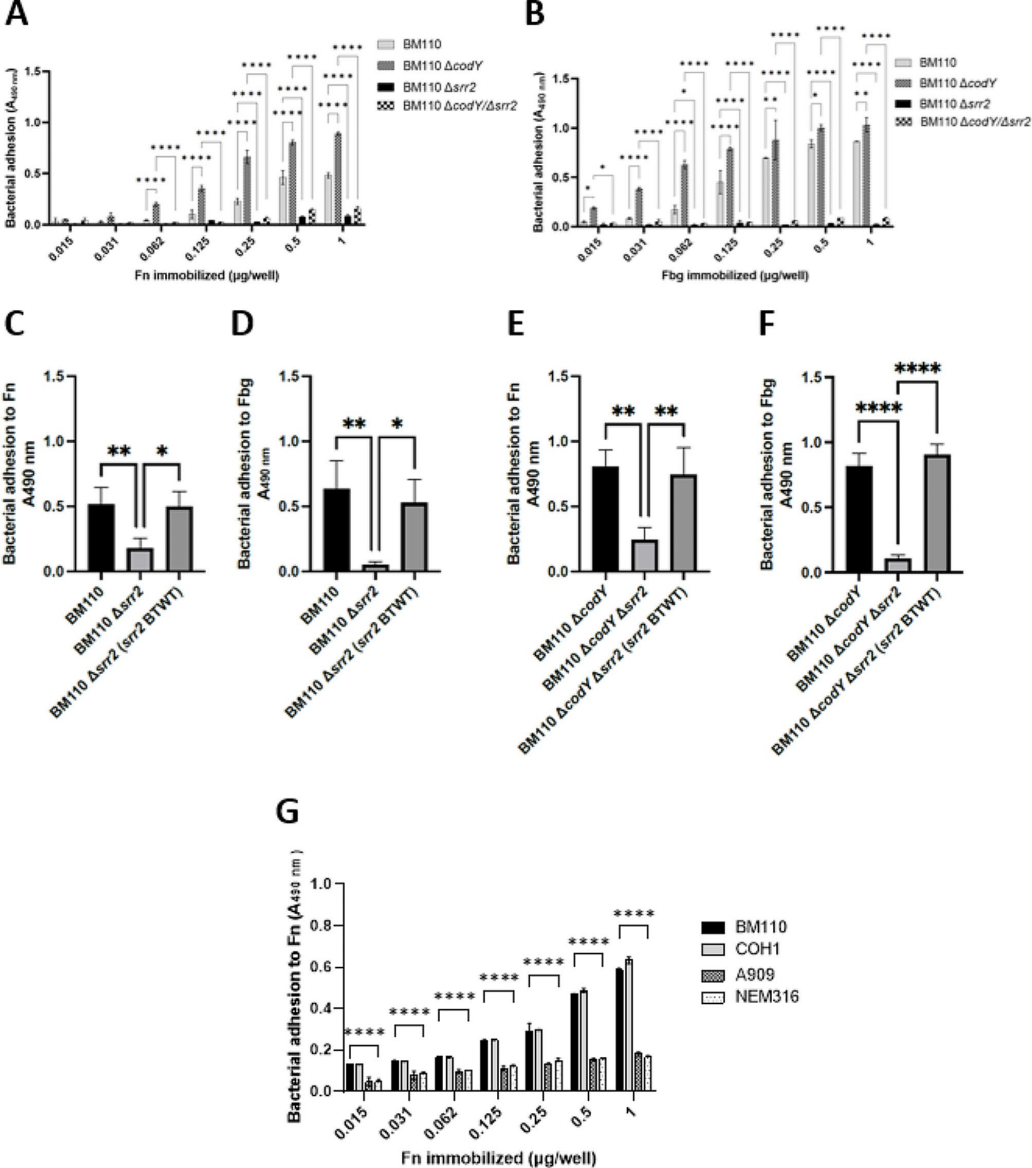
Srr2 promotes specific binding of GBS BM110 ST-17 to Fn. Panels **A**-**B**. Adhesion of the indicated bacterial strains to immobilized Fn (**A**) or Fbg (**B**). Bound bacteria were detected by the addition of polyclonal anti-GBS IgG and secondary HRP-conjugated IgG to the wells. The ligand concentration given on the x-axis is the concentration of the solution used to coat the wells. Statistically significant difference is indicated (*, *P* < 0,05; ** *P* < 0,01; **** *P* < 0,0001). The data points are the means ± SD from three independent experiments, each performed in triplicate. Panels **C**-**F**. Adhesion of the indicated bacterial strains to immobilized Fn (**C**, **E**) or Fbg (**D**, **F**). Bound bacteria were detected as reported for panels **A** and **B**. Statistically significant difference is indicated (*, *P* < 0,05; ** *P* < 0,01). The data points are the means ± SD from three independent experiments, each performed in triplicate. **G**) Adhesion of GBS strains to increasing amounts of immobilized Fn. Bound bacteria were detected as reported for panels **A** and **B**. The ligand concentration given on the x-axis is the concentration of the solution used to coat the wells. Statistically significant difference is indicated (*, *P* < 0,05; ** *P* < 0,01; **** *P* < 0,0001). The data points are the means ± SD from three independent experiments, each performed in triplicate

To investigate whether *codY* deletion affected the adhesive properties of GBS, an ELISA-based assay was performed to evaluate the adhesion of wild-type and ∆*codY* BM110 cells to the immobilised ECM proteins Fn and Fbg, well-known targets of GBS during the colonisation and infection processes [23]. The data shown in Fig. 3A-B clearly indicate that the ∆*codY* mutant strain displayed a higher binding capacity to both ligands. As Srr2 is already known for its high affinity for Fbg [9], we hypothesized that the enhanced ability of the ∆*codY* strain to bind to this ligand could be due to the overexpression of *srr2* gene, as a result of the removal of the repression exerted by CodY. To confirm this hypothesis and investigate whether Srr2 is involved also in binding to Fn, a marker-less in-frame deletion of *srr2* was introduced in the BM110 strain. As expected, the resulting ∆*srr2* strain showed a significant decrease in GBS binding to Fbg compared to the wild-type strain (Fig. 3B, D). Noteworthy, ∆*srr2* strain also showed a remarkable decrease in GBS binding to Fn (Fig. 3A, C). The latter result is unprecedented and further emphasises the role of Srr2 in driving GBS interaction with ECM proteins. Furthermore, a double deletion mutant (∆*codY*/∆*srr2*) lost its capacity to bind both ligands (Fig. 3A, B, E, F). This confirms that the increased adhesion to Fn and Fbg observed in the ∆*codY* mutant, in comparison to the wild-type strain, can be solely attributed to the overexpression of *srr2* and is not a result of differential expression of alternative factors controlled by the global regulator CodY. As an additional experimental evidence of the importance of Srr2 in both Fbg and Fn binding by GBS, *srr2* complementation successfully restored GBS binding to both ligands in the ∆*srr2* and ∆*codY*/∆*srr2* mutants, bringing the binding levels back to those observed in the wild-type and ∆*codY* strains, respectively (Fig. 3C-F).

Altogether, our data concurrently indicate that Srr2 is required for GBS interaction with both Fbg and Fn and that the increased ability of the ∆*codY* mutant to bind ECM proteins (i.e. Fbg and Fn) is caused by Srr2 overexpression in this strain. Accordingly, while the ST-17 strains BM110 [4] and COH1 [8] efficiently bind Fn in a dose-dependent manner, the non ST-17 strains lacking the *srr2* gene (NEM316 and A909) [8, 21] display a significantly lower ability to bind the same ligand (Fig. 3G).

### Characterization of Fn-binding activity of Srr2

**Fig. 4:**
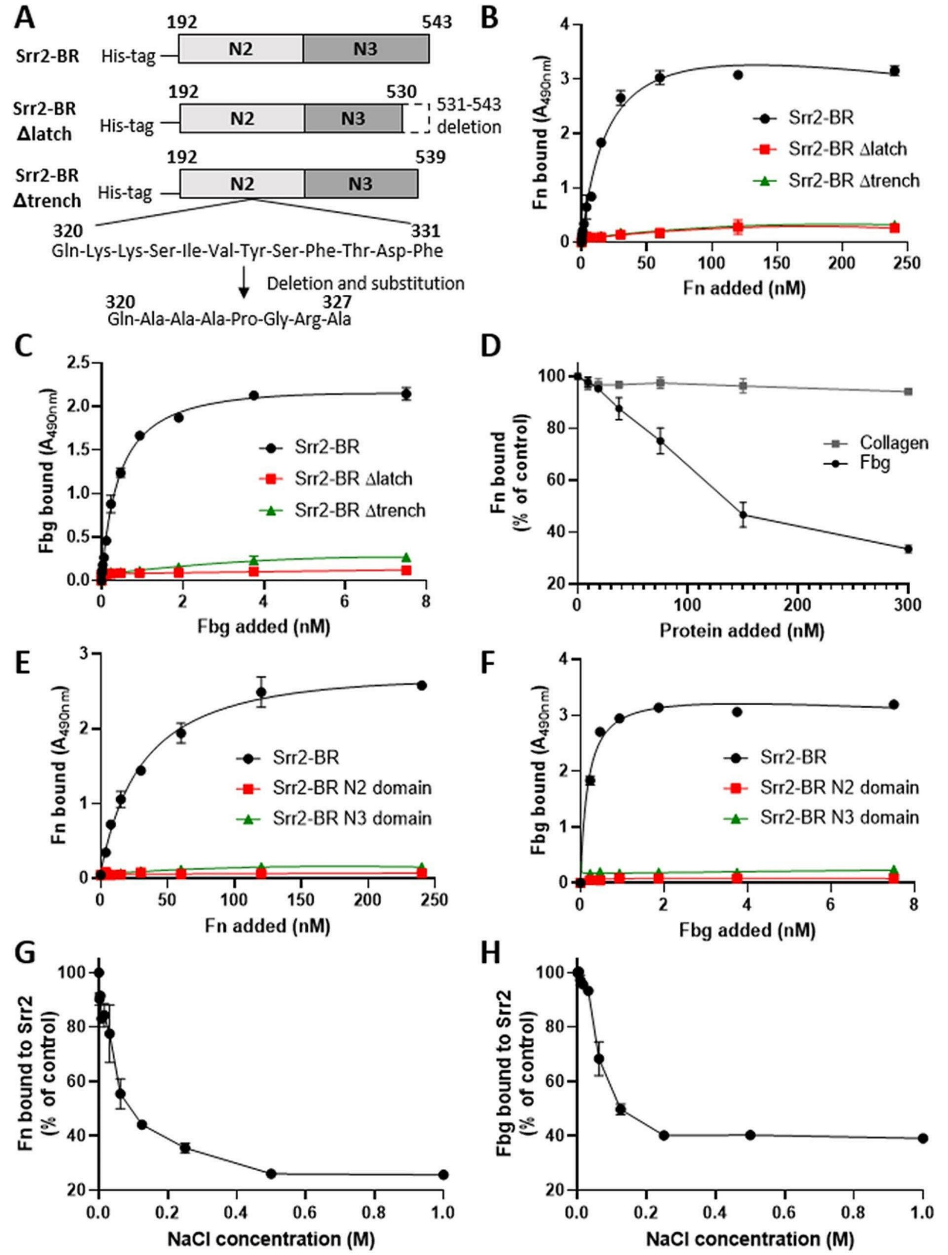
Srr2 binds Fn through the dock-lock and latch mechanism. **A**) Schematic representation of wild-type or mutated recombinant Srr2 binding regions. Srr2-BR is composed of two domains (N2-N3) spanning residues 192 (amino-terminal domain) − 543 (carboxyl-terminal domain). Srr2-BR Δlatch is composed of two domains (N2-N3) spanning residues 192–530 lacking the latching sequence (dotted white box residues 531–543) as previously reported [9]. Srr2-BR Δtrench is composed of two domains (N2-N3) spanning residues 192–539 where the sequence known to form the ligand-binding trench (residues 320–331 in the N2 domain) was replaced with the sequence Gln-Ala-Ala-Pro-Gly-Arg-Ala (residues 320–327) as previously reported [11]. Panel **B**-**C**. Concentration dependent-binding of Fn (**B**) or Fbg (**C**) to immobilized Srr2-BR (black line), Srr2-BR Δlatch (red line) or Srr2-Δtrench (green line). Complex formation was detected by addition of a polyclonal anti-Fn IgG or a polyclonal anti-Fbg IgG to the wells, followed by secondary HRP-conjugated anti-IgG. **D**) Binding of Fn to immobilized Srr2-BR in the presence of equimolar concentration of Fbg or collagen. A saturating amount of Fn was added along with increasing concentrations of the indicated proteins on immobilised Srr2-BR. Complex formation was detected by addition of a polyclonal anti-Fn IgG followed by secondary HRP-conjugated anti-IgG. Data are expressed as a percentage of the control, i.e. incubation performed in the absence of any potential Fn competitor. Panels **E**-**F**. Concentration dependent-binding of Fn (**E**) or Fbg (**F**) to immobilized Srr2-BR (black line) or Srr2-BR N2 (red line) or Srr2-BR N3 (green line). Complex formation was detected as for panels **B**-**C**. Panels **G**-**H**. Binding of Fn (**G**) or Fbg (**H**) to immobilized Srr2-BR was analyzed under increasing concentrations of NaCl. Complex formation was detected as for panels **B**-**C**. Binding data are expressed as a percentage of the control, i.e. incubation performed in the absence of NaCl. Data points are the means ± SD from three independent experiments, each performed in triplicate

To investigate the specificity of Srr2 binding to Fn, the recombinant Srr2 binding region (rSrr2-BR) was produced (Fig. 4A), immobilised onto microtiter wells and tested for binding to increasing concentrations of Fn or Fbg (used as control). The amount of Fn or Fbg bound to immobilized rSrr2-BR was detected using in-house produced polyclonal anti-Fn or anti-Fbg antibodies and a secondary HRP-conjugated antibody. As reported in Fig. 4B, soluble Fn bound the surface-coated rSrr2-BR in a concentration-saturable manner with an apparent K_d_ of 30 ± 1.3 nM. Soluble Fbg bound rSrr2-BR with a higher affinity compared to Fn, with an apparent K_d_ of 0.45 ± 0.02 nM (Fig. 4C), comparable to the affinity previously estimated by others [9]. Of note, rSrr2-BR is also able to bind immobilised Fn although with a 20-fold higher apparent K_d_ (Fig. S2).

To test whether Fbg and Fn share the same binding site on Srr2, rSrr2-BR was immobilised on microtiter wells and the binding of Fn was studied in the presence of increasing concentrations of Fbg (up to 10 μg). Under these conditions, Fbg was able to inhibit Fn binding to rSrr2-BR in a dose-dependent manner up to 60% (Fig. 4D). No inhibitory effect was observed when the assay was conducted in the presence of collagen, used as control. These results suggest that Fn and Fbg share at least in part the same binding site on rSRR2-BR.

Seo et al. demonstrated that Fbg binds Srr2 at the level of the BR exploiting the so-called dock, lock and latch (DLL) mechanism [9]. To the best of our knowledge, the DLL mechanism has never been described for the binding of Fn to bacterial adhesins. Considering the inhibitory effect of Fbg in Fn binding to rSrr2-BR, we investigated whether the inhibition could be due to a steric hindrance or physical binding competition at the same binding site on rSrr2-BR. To study the Fn binding mechanism, we prepared two mutant versions of rSrr2-BR. The rSrr2-BR ∆latch version carries a deletion of the last 12 amino acids of the N3 subdomain, corresponding to the latch fragment involved in host ligand blocking. The rSrr2-BR ∆trench version has a mutation at the level of the docking cleft on the N2 subdomain, where the host ligand is supposed to bind as the first step of the DLL mechanism (Fig. 4A) [11]. Both mutated versions of rSrr2-BR were unable to bind Fn and Fbg (Fig. 4B, C), supporting the hypothesis of a DLL binding mechanism for Fn as well. To further prove the binding mode of Srr2 to Fn, the subdomains N2 and N3 of the Srr2-BR were produced individually, immobilised onto microtiter wells and tested for Fn and Fbg binding. As reported in Fig. 4E, F, the two subdomains lost the ability to bind Fn and Fbg when tested separately. These results indicate that the binding of Fn to Srr2 requires the presence of both N2N3 domains as predicted by the DLL binding model.

To investigate whether ionic forces play a role in the interaction of Srr2 with Fn, the effect of sodium chloride (NaCl) on Fn binding to rSrr2-BR was investigated using the same immunochemical methods highlighted above. The addition of increasing concentrations of NaCl strongly impaired the binding of Fn (Fig. 4G) and Fbg (Fig. 4H) to immobilised rSrr2-BR, furtherly supporting the hypothesis that Fn and Fbg share a similar binding mechanism to Srr2.

### Quantitative assessment of Srr2 binding to Fn by SPR

**Fig. 5:**
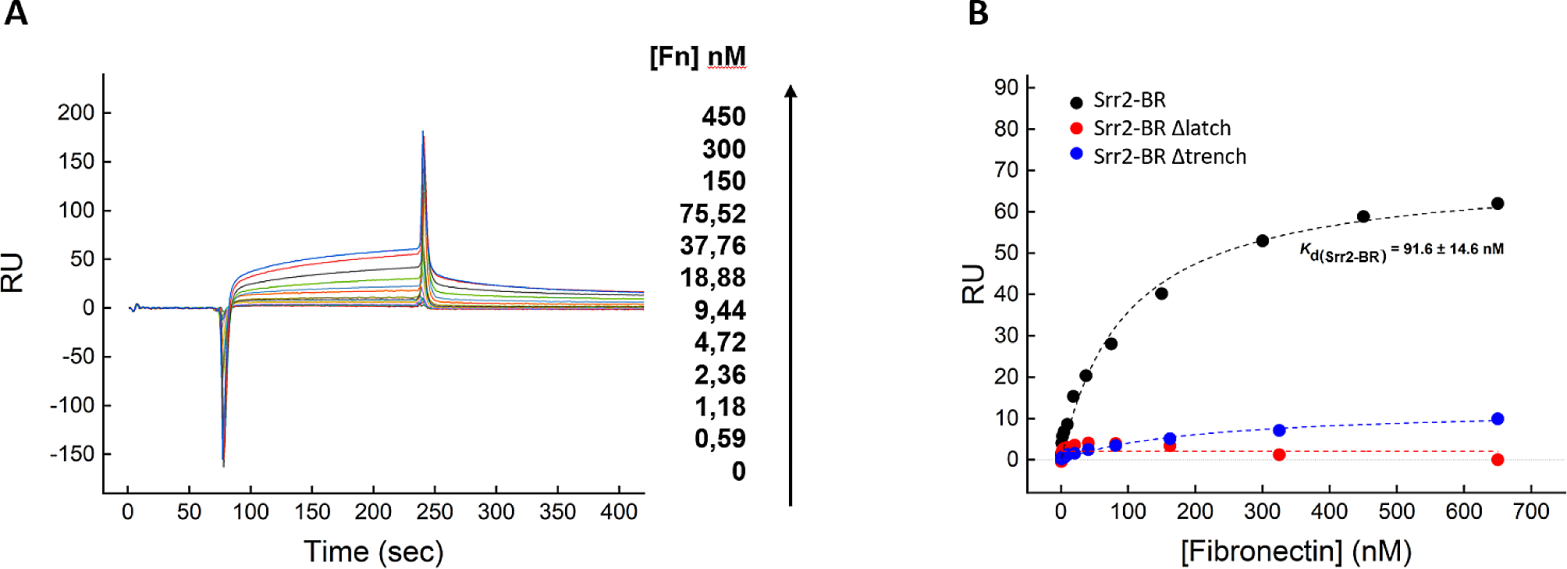
SPR analysis of Fn binding to immobilized rSrr2. Recombinant Srr2 proteins were immobilized onto a CM5 sensor chip and increasing concentrations of fibronectin were injected in the mobile phase. (**A**) Representative sensograms relative to the binding of Fn to Srr2-BR. (**B**) Plot of RUmax vs. Fn concentration relative to the binding to Srr2-BR (●), Srr2-BR ∆latch (●), and Srr2-BR ∆trench (●). Fitting of data points with Eq. 1 yielded the Kd value for the Srr2-BR-Fn complex, as reported. All SPR measurements were carried out at 25 °C in HBS-EP+

A more quantitative estimate of Fn interaction to Srr2 proteins was obtained using a label-free biophysical technique, such as surface plasmon resonance (SPR), yielding a direct measure of the affinity between two interacting partners [19]. For this analysis, each rSrr2 protein was covalently immobilized onto a carboxymethylated-dextran sensor chip (CM5) and increasing Fn concentrations were injected into the mobile phase (Fig. 5). Data analysis was carried out in the affinity mode [24], whereby the SPR signal at the steady state in each sensogram was plotted as a function of Fn concentrations. The resulting data points were interpolated with Eq. 1, describing a one-site binding model [25], to yield an equilibrium dissociation constant (*K*d ) of 92 ± 15 nM for the binding of Fn to immobilized rSrr2-BR, which is comparable with the affinity estimated by ELISA (*K*d = 30 ± 1.3 nM), as reported in Fig. 4B. Furthermore, as already observed with ELISA measurements (Fig. 4B), the binding of Fn to Srr2 Δlatch and Δtrench mutants was almost entirely abolished (Fig. 5B), thus further supporting the hypothesis that Srr2 follows a DLL mechanism in Fn binding.

### Identification of the Fn region bound by Srr2-BR

**Fig. 6:**
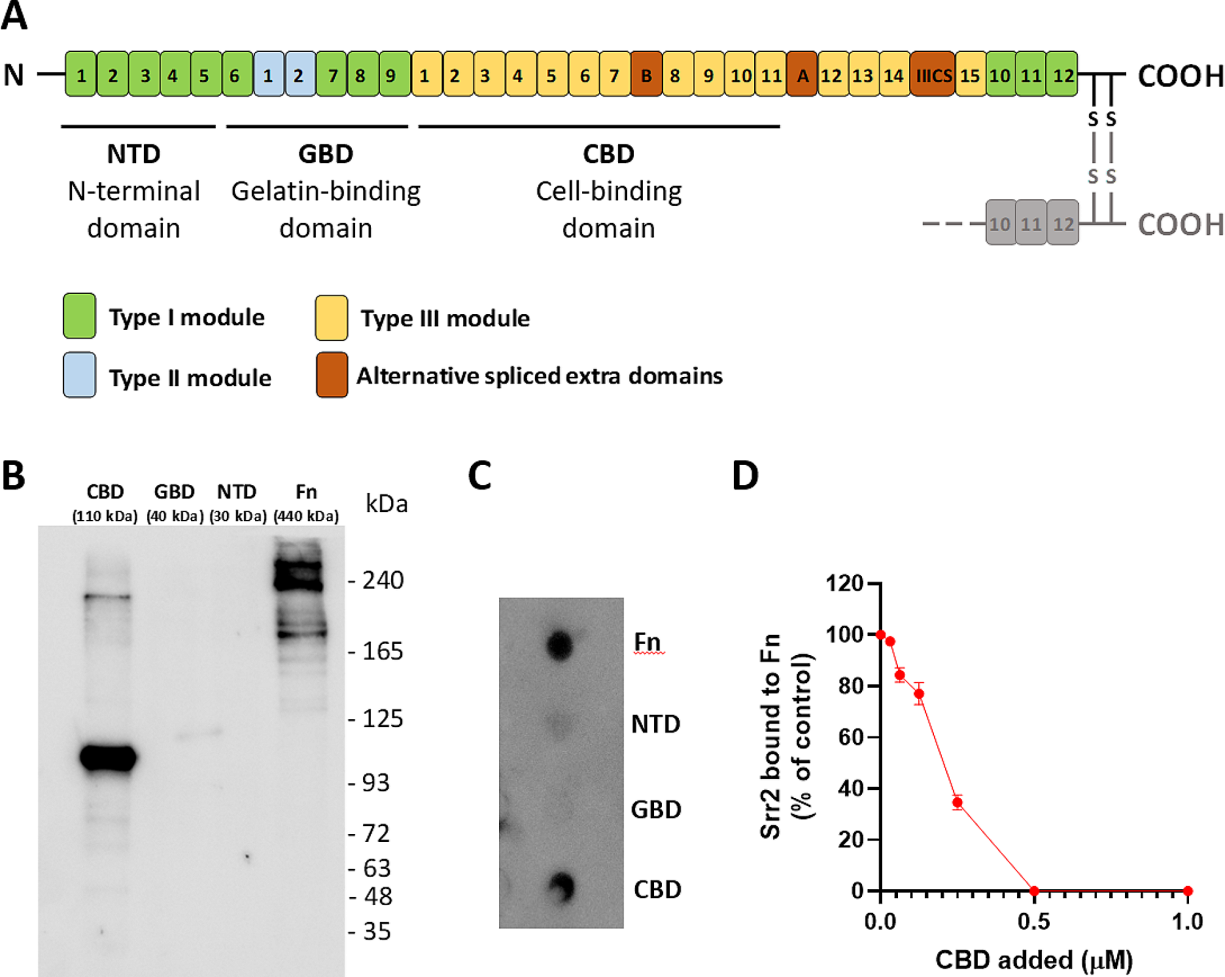
Srr2-BR binds Fn at the level of CBD domain. (**A**) Schematic representation of Fn. Type I, II, and III modules are denoted by green square, light blue square, and yellow square, respectively. Alternative spliced extra domains (**B**-**A** and IIICS) are shown in brown square. N-terminal domain (NTD), the gelatin-binding domain (GBD) and the cell-binding domain (CBD) are also indicated. (**B**) Western-blot analysis of the binding of Fn or its fragments (CBD, 110 kDa; GBD, 40 kDa, and NTD, 30 kDa) to Srr2-BR-conjugated microspheres. After incubation of equimolar concentrations of Fn or its fragments with Srr2-BR-conjugated microspheres, the beads were washed and the complex dissociated using SDS-containing buffer. Samples were then subjected to SDS-PAGE and Western Immunoblotting. The membrane was probed with a polyclonal anti-Fn antibody followed by secondary HRP-conjugated anti-IgG. Molecular masses of standard proteins are indicated on the right. Original blot is presented in Supplementary Fig. 3. (C) Dot-blot analysis of the Srr2-BR binding to Fn or its fragments dotted onto a nitrocellulose membrane. The membrane was probed with Srr2-BR followed by incubation with polyclonal anti-Srr2 antibody and secondary HRP-conjugated anti-IgG. The figures **B** and **C** are representative of three independent experiments. (**D**) Inhibitory effect of CBD fragment on the binding of Srr2-BR to immobilized Fn. Srr2-BR was added along with increasing concentrations of CBD to Fn. Complex formation was detected by addition of a polyclonal anti-Srr2 IgG to the wells, followed by secondary HRP-conjugated anti-IgG. Binding data are expressed as a percentage of the control, i.e. incubation performed in the absence of CBD. The data points are the means ± SD from three independent experiments, each performed in triplicate

Fn is a 440 kDa dimeric protein composed of several domains involved in different functions (Fig. 6A) [26]. To localize the Srr2 binding site in Fn, carboxylate microspheres were covalently coated with rSrr2-BR and then incubated with equimolar concentrations of soluble Fn or Fn fragments, i.e. the N-terminal domain (NTD, 30 kDa), the gelatin-binding domain (GBD, 40 kDa), and the cell-binding domain (CBD, 110 kDa). After washing, the beads were incubated with SDS-containing gel loading buffer, to dissociate the Srr2-Fn complex that might have been formed, and the supernatant samples were subjected to SDS-PAGE. Immunoblotting with anti-FN antibody revealed that rSrr2-BR is able to capture soluble Fn and its CBD fragment (Fig. 6B). The results obtained were also confirmed by a dot-blot assay in which Fn and its fragments were immobilised on a nitrocellulose membrane, incubated with rSrr2-BR, and binding finally revealed with an anti-Srr2 antibody (Fig. 6C). Both assays suggest that the binding site of rSrr2-BR on Fn is located in its CBD. Furthermore, the addition of increasing CBD fragments concentrations dose-dependently decreases the binding of rSrr2-BR to immobilised Fn, such that at 0.5 μM CBD, rSrr2-BR binding is completely abolished (Fig. 6D).

### Srr2 mediates GBS adhesion to epithelial cells via fn binding

**Fig. 7:**
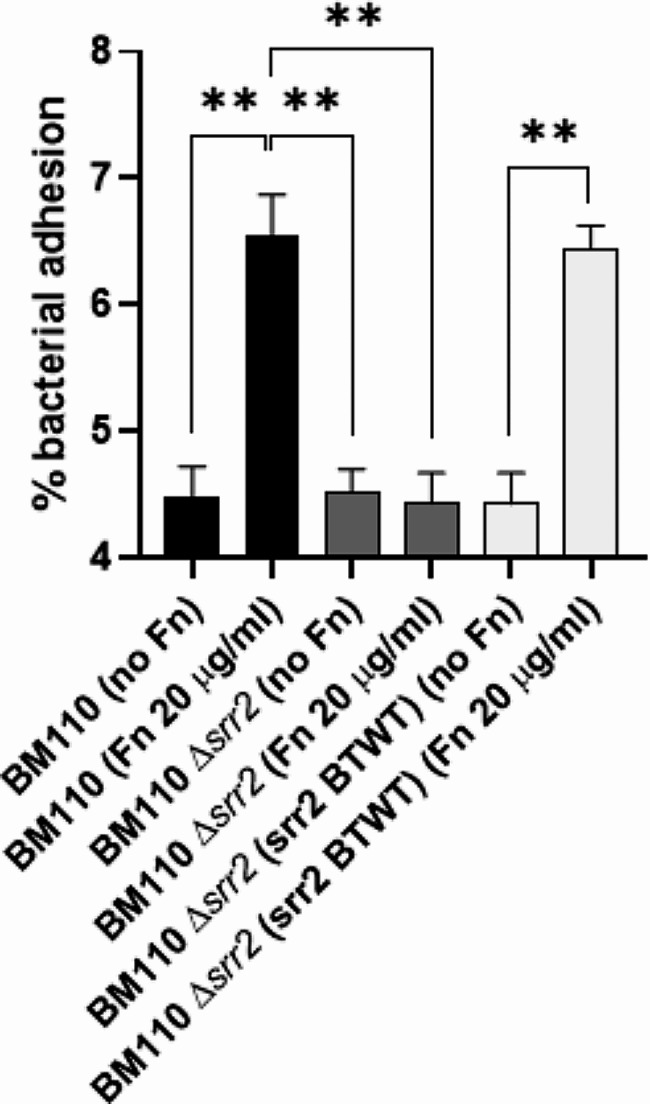
Srr2 mediates GBS adhesion to epithelial cells through Fn binding. Confluent HeLa cells’ monolayers grown in Fn-free medium preincubated or not with exogenously added Fn were infected with the indicated bacterial strains. The percent of adhesion of each strain was calculated relative to the initial inoculum. Statistically significant difference is indicated (** *P* < 0,01). The data points are the means ± SD from three independent experiments, each performed in triplicate

To understand the biological significance of the in vitro results obtained so far in this study, we investigated the possibility that Srr2-Fn interaction could be involved in promoting GBS adhesion to cervico-vaginal epithelial cells. For this purpose, BM110 wild-type or ∆*srr2* cells were used to infect HeLa cell monolayers. The cells were seeded in DMEM medium supplemented with Fn-depleted FBS. The absence of Fn in the monolayer was confirmed by ELISA assay (data not shown). Under these conditions, after infection, no differences in adhesion levels were observed between the wild-type and ∆*srr2* strains (Fig. 7). The addition of exogenous Fn to the cell monolayers prior to infection promoted a significant increase in adhesion of the wild-type but not of the ∆*srr2* strain (Fig. 7). As additional experimental evidence of the importance of Srr2-Fn interaction in mediating GBS adhesion to epithelial cells, *srr2* complementation restored the adhesion levels back to those observed in the wild-type strain (Fig. 7). These results suggest that the interaction of GBS with Hela cells is promoted by the presence of Fn and that this interaction is mediated by Srr2. We also investigated whether the Srr2-Fn interaction could be involved in mediating the invasion of HeLa cells by GBS. Contrary to what was observed for adhesion, no increase in invasion levels was observed in presence of Fn in both wild-type and ∆*srr2* strains, suggesting that the interaction between Srr2 and Fn does not affect BM100 invasion of Hela cells (data not shown).

## Discussion

Fn is widely abundant in animal tissues and fluids and potentially serves as a substrate for bacterial adhesion and host cell infection [27]. Bacteria can express several receptors on their surface, which exhibit recognition specificity towards distinct Fn domains, mainly the N-terminal (NTD) and central cell-binding domains (CBD) [27]. In many cases, Fn receptors engage in functions beyond simple adhesion. In fact, adhesion is often the prerequisite for the invasion and internalisation of microorganisms into the cells of colonised tissues. Furthermore, the presence of Fn on the surface of microbes may enable them to evade recognition by host defence mechanisms [27]. Consequently, Fn-binding proteins on bacterial surfaces can serve both as adhesion/invasion and anti-opsonic factors, and therefore have good potential as virulence determinants.

Among the best-characterised Fn adhesins are two proteins encoded by closely related genes, Fn-Binding Protein A and B (FnBPA and FnBPB), expressed by the majority of *Staphylococcus aureus* strains [28]. FnBPA and FnBPB have the ability to bind the NTD of Fn through an intricately disordered region consisting of several repeating units (11 repeats for FnBPA and 10 for FnBPB) located at the C-terminus of the proteins [29]. Each repeat unit binds Fn with varying affinities, adopting an ordered secondary structure upon binding. At least six repeats bind to the NTD of Fn with high affinity through the β-zipper model [30–32].

Several Fn-binding adhesins have been described in streptococcal species, e.g. *S. pyogenes* [33] and *S. pneumoniae* [34–36]. The Fn binding proteins identified in GBS so far include: (i) the streptococcal C5a peptidase B (ScpB), which is able to interact with adsorbed Fn [37], (ii) the streptococcal Fn binding protein A (SfbA), which facilitates the invasion of GBS into human brain microvascular endothelial cells (hBMECs) in vitro and contributes to meningitis development in vivo [38], and (iii) the GBS bacterial surface adhesin (BsaB), that promotes GBS attachment to epithelial vaginal ME-180 cells [39].

In this work we show that deletion of the global transcriptional regulator CodY in the hypervirulent ST-17 GBS strain BM110 causes a significant increase in bacterial interaction with Fn and Fbg. We have recently shown that deletion of *codY* increases the expression of the *srr2* gene [15]. Considering that this adhesin is a well-known ligand of Fbg [9], we postulated its potential involvement also in Fn binding. The results reported in this study concurrently support this hypothesis, as the deletion of *srr2* resulted in a remarkable decrease in the ability of strain BM110 to bind not only Fbg (used as control) but also Fn. Conversely, complementation of the *srr2* mutation restored binding to wild-type levels, strongly suggesting that the protein can indeed play a role in this interaction. As deletion of *srr2* in the *codY-null* background significantly impaired bacterial binding to Fn, we ruled out the possibility that other proteins, whose expression is controlled by the global regulator CodY, could account for the increase in Fn binding observed in the *codY* mutant strain when compared to the wild-type. Nonetheless, bacterial interaction with Fn was restored in the back to wild-type strain (Δ*codY* Δ*srr2* (*srr2* BTWT)). Noteworthy, a residual binding to Fn was still detectable upon deletion of *srr2*, either in the single or double *Δsrr2/codY* deletion mutants. This could be due to the other Fn-binding adhesins of GBS (e.g. ScpB, SfbA, BsaB), whose expression might be regulated by different mechanisms independent from the regulator CodY [37–39]. Redundancy of bacterial adhesins known to bind the same ligand is common among bacterial pathogens. The extensive redundancy of GBS surface adhesins that bind Fn could be exploited by the bacterium to colonise different host niches, whose environmental signals could influence the expression of an adhesin ahead of another.

The hypervirulent ST-17 strains exhibit a significantly higher interaction with Fn compared to the non-ST-17 strains. Therefore, the capacity of Srr2 to interact with Fn, abundantly found in both plasma and the extracellular matrix, may substantially contribute to the enhanced virulence of ST-17 strains [8].

GBS colonisation of the vaginal mucosa is the very first essential step for bacterial pathogenesis [40]. In this work we observed that in the presence of Fn, GBS shows significantly greater adhesion to cervico-vaginal epithelial (HeLa) cell monolayers. Conversely, the *srr2* deletion mutant exhibited comparable adhesion levels to epithelial cell monolayers regardless of the presence or absence of Fn. Our results indicate that the interaction between Srr2 and Fn contributes to cell adhesion but not to the invasion process for the cell line under our investigation. However, we cannot exclude that it is involved in invasion of other cell lines. It is likely that Srr2 binding to Fn is important for GBS colonisation and niche establishment in the vaginal tract. As the most common route of GBS dissemination to the newborn is by vertical transmission at the moment of labour from a vaginally colonised mother, we can speculate that Srr2 interaction with Fn at the level of the vaginal epithelium could additionally contribute to the increased pathogenic potential of GBS ST-17 strains. Furthermore, the Srr2-Fn interaction could provide additional insight into the greater adhesive properties of ST-17 strains compared to non-ST-17 isolates [4].

These isolates are able to translocate across the intestinal epithelial barrier via transcytosis of Peyer’s patch M cells through Srr2 [7]. It is interesting to note that Fn is abundantly expressed in the intestinal epithelium [41]. Consequently, the interaction between Srr2 and Fn might facilitate gut colonisation by GBS ST-17 strains, serving as an initial step that promotes subsequent stages of bacterial invasion in the host. In a future study, it would be worth investigating the role of Srr2-Fn interaction in the colonisation of the intestinal epithelium by strain ST-17.

In order to deepen our understanding of Srr2-Fn interaction at the molecular level, we produced by recombinant DNA techniques the BR domain of Srr2, which has been already known to interact with Fbg [9]. The results obtained provide solid experimental evidence that the BR region is involved in Fn binding as well. The apparent Kd value of the Srr2-BR-Fn interaction obtained by ELISA was approximately 60-fold higher than that measured for Fbg. It is conceivable that the interaction between Srr2 and Fn plays a greater role in Fn-rich tissues. Srr2-Fn interaction was also confirmed by surface plasmon resonance, obtaining a Kd value comparable to that determined by ELISA. Interestingly, we observed that rSrr2-BR is able to bind both soluble and immobilised Fn. This property differs from other GBS Fn-binding proteins, such as rScpB, able to bind only immobilised Fn [37]. This ability could influence the colonisation and pathogenesis of GBS strains expressing Srr2.

The strong inhibition exerted by Fbg on the binding of Fn to the Srr2-BR region suggests that the binding sites for the two proteins on the Srr2-BR region are partially overlapped. Furthermore, increasing ionic strength had a detrimental effect on the binding of both Fn and Fbg to Srr2, indicating that electrostatic interactions play a significant role in the formation of these complexes. These results led us to assume that both ligands exploit a similar mechanism to bind the BR region. Since the binding of Fbg to the BR region has previously been shown to occur *via* the dock, lock, and latch (DLL) [9] mechanism, here we decided to verify whether this mechanism was also involved in the interaction with Fn. We found that Fn binds to the BR region of Srr2, most likely by the DLL mechanism used to bind Fbg, because variants lacking the ability to bind Fbg were also defective in Fn binding. To localise the binding site(s) of Srr2-BR on Fn, we tested its interaction with the main Fn fragments. The results obtained indicate that Srr-BR binds Fn at the level of the cell-binding domain (CBD). Similar conclusions have been also reported for other Fn-binding bacterial adhesins, such as BBK32 from *Borrelia burgdorferi* [42] and Scl1 from *S. pyogenes* [43]. In our laboratory, structural biology studies are underway to locate which of the type III modules, forming the Fn CBD domain, are involved in the interaction with Srr2-BR. Whereas the results of this study concurrently indicate that Srr2-BR is the hot spot for both Fbg and Fn binding, the structural and physico-chemical properties of the putative Srr2 binding regions on the two proteins remain elusive.

Intriguingly, Fbg and Fn display remarkably different structural properties and organization, but nevertheless, our results concurrently indicate that they are both able to interact with the same target adhesin (i.e., Srr2), at roughly the same binding site (i.e., Srr2-BR), and exploiting the same interaction mechanism (i.e., DLL).

More specifically, Fbg consists of three non-identical polypeptide chains (Aα-, 610 residues; Bβ-, 461 residues; γ-, 411 residues in the human species) and circulates in the plasma as a rod-shaped dimer of the three chains (AαBβγ)_2_, held together by disulfide bridges and assembled in a three-nodule architecture, with a central “E” nodule formed by the N-termini of the six chains while the C-termini of the Bβ and γ chains extend outward to form two distal “D” nodules [44]. The region between the E and D nodules is mainly a three-stranded α-helical coiled-coil connector, fixed by disulfide bonds, while the Aα chain extends with the flexible αC connector region (amino acids 221–391) and the two structured/globular αC domains (amino acids 392–610). The flexible αC connector contains ten 13-residues tandem repeats, all rich in proline and glycine [45]. Noteworthy, earlier work has localized the binding site for Srr2 on Fbg structure at the level of the tandem repeats 6–8 of the conformationally flexible αC connector [9]. Conversely, Fn is a dimeric protein made of two 250-kDa subunits, which are linked by a pair of disulfide bonds at their C-terminal ends [46]. Two forms of Fn are known, i.e. a plasma form, predominantly synthesized by liver hepatocytes, and a cellular form, which is produced by a wide variety of cells including fibroblasts, chondrocytes, myocytes, and synovial cells. Plasma Fn circulates in the blood, while cellular Fn is locally secreted in the ECM. The molecule is composed of globular modules of type I, II and III, each containing two sandwiched β-sheets and connected in series to provide a very long contour length of 120–160 nm and the sites for Fn-binding proteins [47]. Whereas type-I and type-II modules each contain two disulfide bonds that structurally reinforce the domain fold, type-III modules do not have any internal disulfide bonds, highlighting the possibility that these domains could be stretched by mechanical forces for interacting with other proteins [48]. The binding data reported in this study show that Srr2 interacts with the CBD, mainly formed by type-III modules, puts forward the promiscuity of Srr2 in protein binding, and emphasizes the structural plasticity that Srr2-BR should have to recognize different protein entities, such as Fbg and Fn. Structural biology studies are awaited to address this key feature of Srr2 interaction mechanism.

In conclusion, the results of this work highlight a new role for the multivalent adhesin Srr2 expressed by the hypervirulent strain BM110. Noteworthy, the role of Srr2 interaction with Fn in promoting GBS adhesion to epithelial cells could represent a novel pathogenic route that could explain the increased virulence potential of this strain in the development of GBS-related diseases. Further experiments, however, are awaited to validate Srr2-Fn binding complex as a druggable interface for the development of novel therapeutic strategies aimed at interfering with GBS adhesion to target tissues and, finally, in the treatment of GBS infections.

### Electronic supplementary material

Below is the link to the electronic supplementary material.

Supplementary Material 1

Supplementary Material 2

Supplementary Material 3

## Data Availability

Data is provided within the manuscript or supplementary information files.
