# Supplementary material for: The serine-rich repeat glycoprotein Srr2 mediates Streptococcus agalactiae interaction with host fibronectin
Source: BMC Microbiol. 2024 Jun 22;24:221. doi: 10.1186/s12866-024-03374-6 (PMC11193222; doi:10.1186/s12866-024-03374-6)
Supplement: Supplementary file 1 — Supplementary Material 1 [file 12866_2024_3374_MOESM1_ESM.pdf]

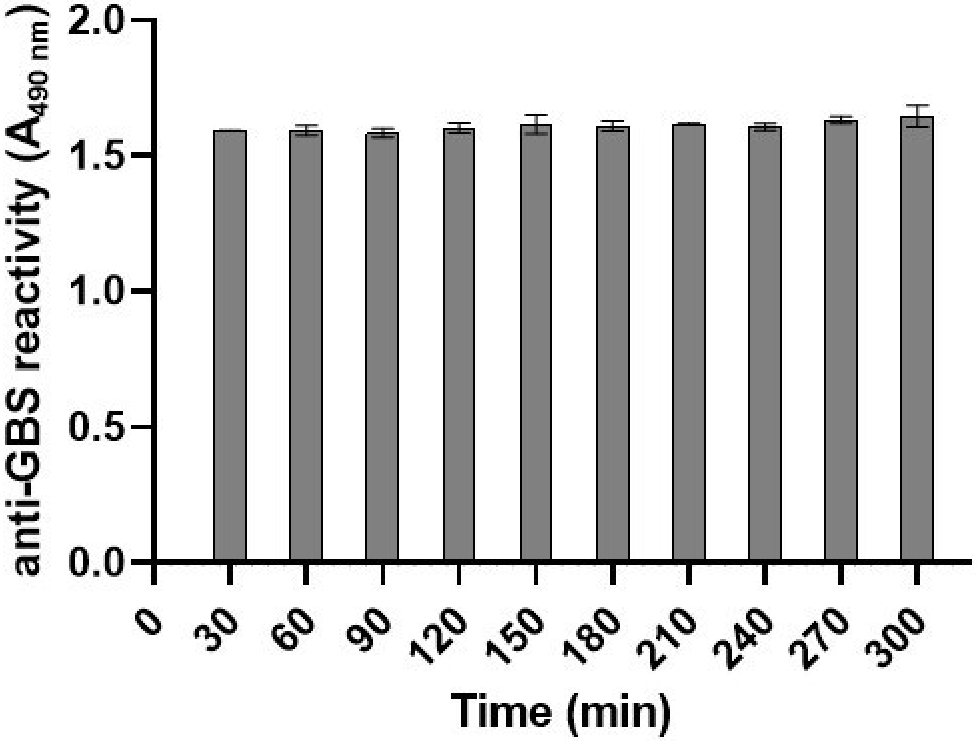

Supplementary Fig. 1. The amount of BM110 cells immobilised is identical at the different time points. At the indicated times, bacterial cells were collected, washed and then immobilized onto microtiter wells. The presence of the same amounf of cells immobilized was revealed using an anti-GBS antibody, followed by secondary HRP-conjugated IgG. The data points are the means ± SD from three independent experiments, each performed in triplicate.
