# Supplementary material for: The serine-rich repeat glycoprotein Srr2 mediates Streptococcus agalactiae interaction with host fibronectin
Source: BMC Microbiol. 2024 Jun 22;24:221. doi: 10.1186/s12866-024-03374-6 (PMC11193222; doi:10.1186/s12866-024-03374-6)
Supplement: Supplementary file 2 — Supplementary Material 2 [file 12866_2024_3374_MOESM2_ESM.pdf]

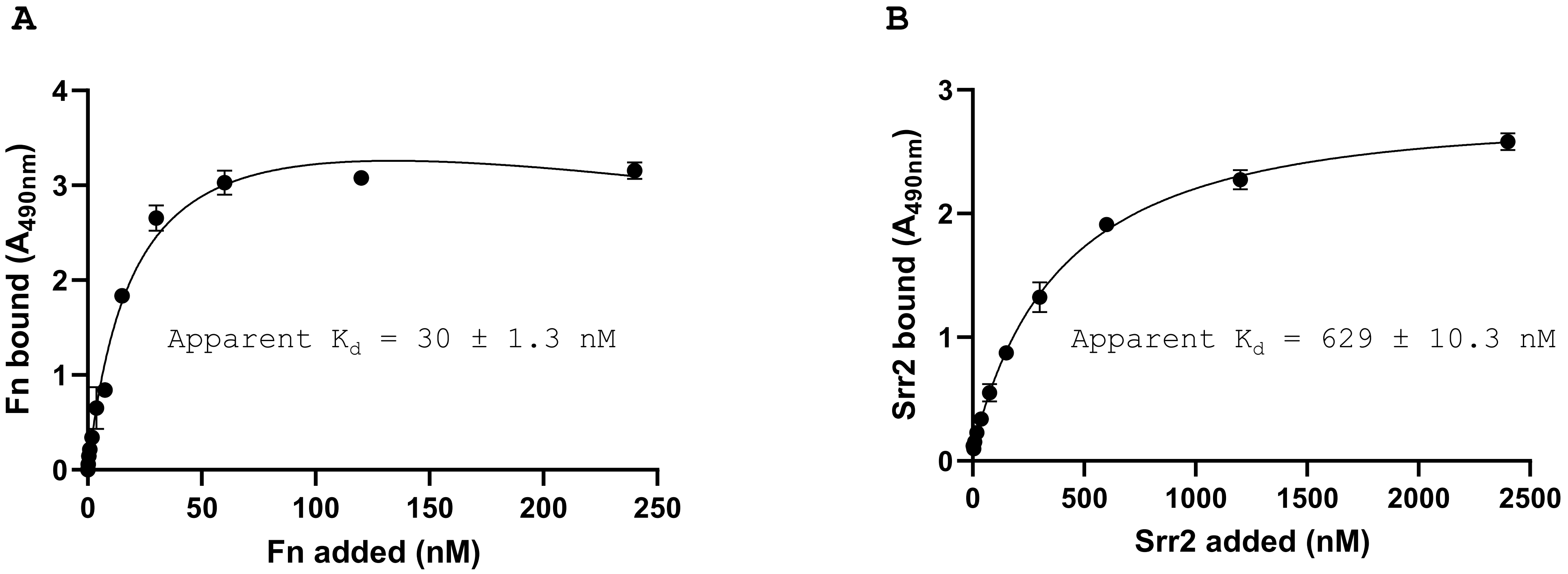

Supplementary Fig. 2. Concentration dependent-binding of Srr2-BR to soluble (A) or immobilized (B) Fn. Complex formation was detected by addition of a polyclonal anti-Fn IgG (A) or a polyclonal anti-Srr2 IgG (B) to the wells, followed by secondary HRP-conjugated anti-IgG.
