# Supplementary material for: The serine-rich repeat glycoprotein Srr2 mediates Streptococcus agalactiae interaction with host fibronectin
Source: BMC Microbiol. 2024 Jun 22;24:221. doi: 10.1186/s12866-024-03374-6 (PMC11193222; doi:10.1186/s12866-024-03374-6)
Supplement: Supplementary file 3 — Supplementary Material 3 [file 12866_2024_3374_MOESM3_ESM.pdf]

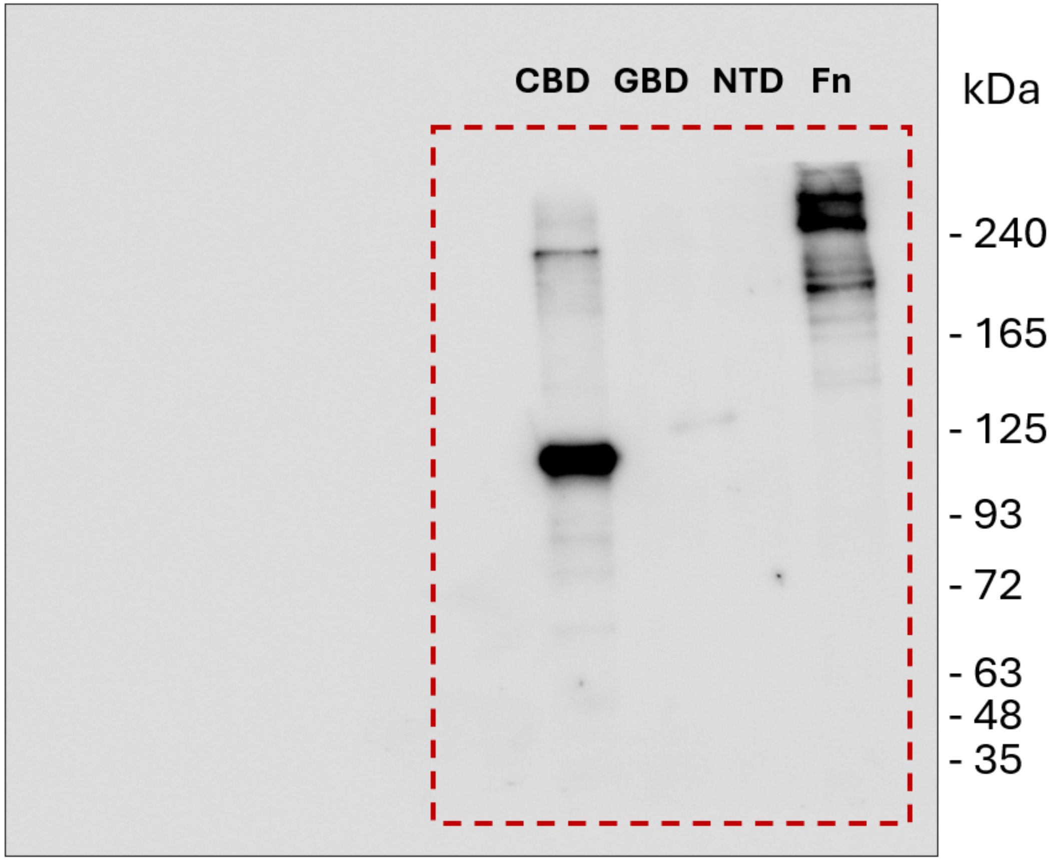

Supplementary Fig. 3. Full-length original Western blot shown in Fig. 5, panel B of the main text is reported. The membrane was slightly cropped on the edges to remove the part where no samples were loaded. The area of the cropped membrane is indicated by a red dashed line. The standard expected molecular weight or protein size markers are reported on the right.
